# Development of Tailor-Made Dendrimer Ternary Complexes for Drug/Gene Co-Delivery in Cancer

**DOI:** 10.3390/pharmaceutics13081256

**Published:** 2021-08-13

**Authors:** Ana Raquel Neves, Tânia Albuquerque, Rúben Faria, Milan Paul, Swati Biswas, Ângela Sousa, Diana Costa

**Affiliations:** 1 CICS-UBI—Health Sciences Research Centre, University of Beira Interior, Avenida Infante, D. Henrique, 6200-506 Covilhã, Portugal; ana.neves@ubi.pt (A.R.N.); tania.albuquerque@ubi.pt (T.A.); ruben_faria95@hotmail.com (R.F.); angela@fcsaude.ubi.pt (Â.S.); 2 Nanomedicine Research Laboratory, Department of Pharmacy, Birla Institute of Technology & Science—Pilani, Hyderabad Campus, Jawahar Nagar, Medchal, Hyderabad, Telangana 500078, India; milanpaul@hyderabad.bits-pilani.ac.in (M.P.); swati.biswas@hyderabad.bits-pilani.ac.in (S.B.)

**Keywords:** cancer therapy, cellular uptake, dendrimers, drug/gene co-delivery, ternary delivery systems

## Abstract

Cancer gene therapy, mediated by non-viral systems, remains a major research focus. To contribute to this field, in this work we reported on the development of dendrimer drug/gene ternary complexes. This innovative approach explored the great capacity of both polyamidoamine (PAMAM)-paclitaxel (PTX) conjugate and polyethylenimine (PEI) polymers to complex a p53-encoding plasmid DNA (pDNA), highlighting the utility of considering two compacting agents. The pDNA complexation capacity has been investigated as function of the nitrogen to phosphate groups ratio (N/P), which revealed to be a tailoring parameter. The physicochemical properties of the conceived ternary complexes were revealed and were found to be promising for cellular transfection. Furthermore, the formulated co-delivery systems demonstrated to be biocompatible. The ternary systems were able of cellular internalization and payload intracellular release. Confocal microscopy studies showed the co-localization of stained pDNA with the nucleus of cancer cells, after transfection mediated by these carriers. From this achievement, p53 gene expression occurred with the production of protein. Moreover, the activation of caspase-3 indicated apoptosis of cancer cells. This work represents a great progress on the design of dendrimer drug/gene co-delivery systems towards a more efficient cancer therapy. In this way, it instigates further in vitro studies concerning the evaluation of their therapeutic potential, expectedly supported by the synergistic effect, in tumoral cells.

## 1. Introduction

Despite the great progress in cancer research, this disease is still the second leading cause of death worldwide, according to the World Health Organization. Recent innovative strategies to deal with this severe disease include immunotherapy, thermal ablation of tumors, magnetic hyperthermia, targeted therapy or gene therapy [1]. The field of gene therapy has been extensively explored in recent decades as a potential treatment for cancer, as it focuses on the actual cause of the disease. It promotes the expression or silencing of a specific gene to achieve a therapeutic effect [2,3]. Gene therapy based on the reestablishment of p53 tumor suppressor protein has been one of the most researched approaches, as TP53 gene is mutated in, about, 50% of all human cancers [4,5]. p53 plays a relevant role in cell apoptosis and in the regulation of cell cycle, being critical for normal cell function [6].

Viral vectors, such as retrovirus or adenovirus, represent an effective form of gene delivery and are recognized for their high cellular transfection ability. However, they present several drawbacks that can limit their application. Among the disadvantages of viral vectors are the immune-related toxicity with repeated administration, possible replication competence, non-targeting, or the limited insert size [7,8]. Cancer gene therapy has benefit from the crescent development in the design/formulation of non-viral systems for precise and targeted gene delivery [9,10,11,12]. Non-viral vectors can be easy and tailor-produced, can incorporate a large amount of genetic content, are biocompatible, non-toxic and non-immunogenic. These synthetic systems have been designed to be stimuli-responsive, tunable and targeted, while promoting controlled and sustained gene release [13,14,15]. Moreover, the simultaneous release of genes and anticancer drugs to cancer cells, mediated by non-viral carriers, demonstrated to enhance the anticancer therapeutic effect [9,16,17,18]. In this sense, micelles, lipid, peptide and polymer-based nanoparticles have been developed to promote cellular uptake, endosomal escape, specific organelle targeting, gene delivery and, ultimately, therapeutic effect [19,20,21,22]. Due to their chemical structures and the versatility to modify them, their tailored architectures, along with biodegradable and bio-reducible properties, among others, polymers represent a suitable platform to load/encapsulate genes and anticancer drugs [23,24,25]. Among polymers, dendrimers hold remarkable characteristics for payload delivery [26,27]. Dendrimers are globular molecules, with three-dimensional structures composed by a central core with multiple repeating units covalently linked to the nucleus (referred as generations, G), a hyperbranched mantle and a corona bearing reactive functional groups. The great control over the synthesis of their architectures turns dendrimers into nanometer carriers with predictable and tailored properties [28,29]. Therefore, dendrimers are monodisperse and present remarkable chemical and physical stability. Additionally, different functional groups can be added to their surfaces allowing bioactive molecules such as anticancer drugs, genes, signaling molecules or antibodies to be attached, what may significantly contribute to enhance the delivery capacity of dendrimers. Several types of dendrimers, including PAMAM, poly-L-lysine, or poly(propylene imine), have been conveniently studied for biomedical applications [26,30]. PAMAM which have ethylenediamine core, amine and repetitive amide bonds in the periphery, are highly accessed dendrimers for drug delivery. The number of primary amine groups on the surface of dendrimers could efficiently condense nucleic acids. Along with acting as a gene delivery vector, the well-defined core in the PAMAM dendrimer could encapsulate hydrophobic anticancer drugs or conjugate the drug on the surface [31]. PAMAM dendrimers are one of the most widely considered molecules for targeted drug and gene delivery, due to a set of favorable properties as, for instance, the possibility of core and surface groups modification, their hydrophilic nature, and the great biocompatibility [29,32]. Both in vitro and in vivo studies have confirmed the efficacy of PAMAM-based delivery systems to achieve the target site and release the therapeutic payload [33,34]. Despite the relevant advances achieved on the research focused on dendrimers for biomolecules delivery towards cancer therapy, their clinical translation has not progressed at the same level.

To contribute for advances on this topic, in this work and for the first time, we combined the great pDNA condensation capacity exhibited by both PAMAM and PEI polymers to create drug/gene ternary complexes. Our strategy of using two pDNA compacting agents revealed to be successful, as pDNA was almost totally complexed. Moreover, the developed ternary complexes revealed to possess adequate physicochemical characteristics for drug/gene co-delivery. Additionally, these properties can be tailored by varying the N/P ratio parameter. The systems were easily internalized into cancer cells and were able of nucleus co-localization. Following this, p53 gene could be expressed and the protein produced. First studies on the evaluation of therapeutic effect, mediated by the developed complexes, seemed to indicate cancer cells apoptosis. To the best of our knowledge, the new dendrimer drug/gene PEI complex is a novelty and represents a promising nano-platform to be further investigated for anticancer therapy.

## 2. Materials and Methods

### 2.1. Materials

Fourth-generation dendrimer having surfaced amino groups and ethylenediamine core groups (PAMAM G4) was procured from Dendritech (Midland, MI, USA). Paclitaxel (PTX) was kindly provided as a gift sample by Fresenius Kabi India Pvt., Ltd. (Gurgaon, India). *N*-(3-Dimethylaminopropyl)-*N*-ethyl carbodiimide hydrochloride (EDC. HCl, 98%) and *N*-hydroxysuccinimide were obtained from Sigma Aldrich Chemicals (St. Louis, MO, USA). Cellulose dialysis membrane of MWCO 3.5 kDa was purchased from Spectrum Laboratories, Inc. (Dominguez, CA, USA). All the other chemicals and solvents obtained commercially were of analytical grade. Commercial branched PEI with average Mw 25 kDa, 3-(4,5-dimethylthiazol-2-yl)-2,5-diphenyltetrazolium bromide (MTT) and fluorescein isothiocyanate (FITC) were obtained from Sigma-Aldrich (St. Louis, MO, USA). Agarose and GreenSafe were obtained from NZYtech (Lisbon, Portugal). DAPI was from Invitrogen (Carlsbad, CA, USA). DMEM/F12 cell culture medium was purchased from Corning (New York, NY, USA). All solutions were freshly prepared using ultra-pure grade water, purified with a Milli-Q system from Millipore (Billerica, MA, USA).

Cancer HeLa cells and mouse brain neuroblastoma (N2a) cells were purchased from Invitrogen. Normal Human Dermal Fibroblasts (NHDF) isolated from the dermis of adult skin, Ref. C-12302 (cryopreserved cells) were acquired from PromoCell (Heidelberg, Germany). The 6.07 kbp plasmid pcDNA3-FLAG-p53 was from Addgene (Cambridge, MA, USA; plasmid 10,838) and was purified by a procedure described in the literature [35].

### 2.2. Methods

#### 2.2.1. Synthesis of PTX-2-Hemisuccinate (PTX-SA)

**Scheme 1:**
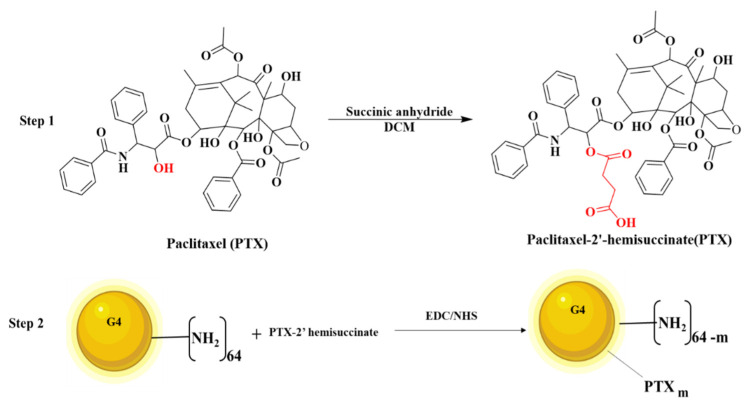
Schematic representation of the synthesis of multifunctional PAMAM G4 dendrimer conjugate.

The synthesis of the conjugate was performed following Scheme 1. The -OH group present on the PTX was activated to hemisuccinate by using succinic anhydride. Briefly, PTX (25 mg) in DCM (2 mL) was added to the succinic anhydride (4.4 mg) at a molar ratio of 1:1.5 under the presence of dry pyridine under continuous stirring. This reaction was carried out for three days [36]. Finally, PTX-2-hemisuccinate was obtained by extracting the product obtained after the reaction using ethyl acetate and vacuum dried to obtain a white crystalline powder.

#### 2.2.2. Synthesis of PAMAM G4-PTX 

The PTX-2-hemisuccinate was activated using EDC/NHS in DMF (2 mL) at room temperature for 6–8 h [37]. Next, the PAMAM G4 dendrimer (50 mg) in DMF was added dropwise to the activated PTX-NHS ester at a molar ratio of 1:4 under the presence of nitrogen. The reaction was carried out overnight, and the DMF was evaporated under vacuum. Finally, the crude reaction mixture was subjected to dialysis for 48 h using a 3500 Da MWCO dialysis membrane. After the dialysis, the product was lyophilized to obtain a fluffy white powder.

#### 2.2.3. Nuclear Magnetic Resonance (NMR) Spectroscopy

The ^1^H-NMR spectra of PAMAM G4, PTX-SA, PAMAM G4-PTX were taken in the solvent CDCl_3_ using a 400 MHz NMR Spectrometer (Bruker, Billerica, MA, USA). The samples (10 mg/mL) were dissolved in the solvent, and the instrument was run with 128 scans.

#### 2.2.4. Preparation of PAMAM G4-PTX/pDNA and PAMAM G4-PTX/pDNA/PEI Complexes

PAMAM G4-PTX was supplied as a lyophilized powder and suspended in DMSO:Water (0.8:0.2) for a final concentration of 10 mg/mL. PEI was suspended in sodium acetate buffer (0.1 mM sodium acetate, pH 4.5) for a final concentration of 5.56 mg/mL. After that, PAMAM G4-PTX and PEI solutions were prepared according to the N/P ratio pretended. Solutions of dendrimers were stored at 4 °C and PEI solutions at room temperature, according to manufacturer’s instructions. For the formulation of PAMAM G4-PTX/pDNA binary complexes at different N/P ratios, variable concentrations of PAMAM G4-PTX were added to a fixed amount of pDNA (1 μg), at vortex for 60 s. To formulate PAMAM G4-PTX/pDNA/PEI ternary systems, 100 µL of PEI (at N/P ratios of 1, 1.5 and 2) were added to a fixed volume of pDNA (1 µg) and then variable concentrations of PAMAM G4-PTX were added to the pDNA/PEI mixture at vortex for 60 s. Both complexes were prepared at various N/P ratios ranging from 2–100, considering the molar ratio of positively charged amine groups (N) from PAMAM G4-PTX to negatively charged phosphates (P) from pDNA. All the systems were left for 30 min at room temperature to allow complexes formation and then centrifuged at 10,000× *g* for 20 min at 4 °C to obtain a pellet containing the pDNA based systems. 

The electrophoretic mobility of the supernatants from all formulations was evaluated by an agarose gel electrophoresis to control if all pDNA amount was complexed. The pDNA complexation capacity (CC) was determined spectrophotometrically measuring the absorbance of the supernatant at 260 nm using a NanoPhotometer™ (Implen, Inc.; Westlake Village, CA, USA). This parameter was determined from the equation:

CC (%) = (Total amount of pDNA-Non-bound pDNA)/Total amount of pDNA (1)

#### 2.2.5. Agarose Gel Electrophoresis

The 1% (*w*/*v*) agarose gel (0.5 g of agarose) was prepared in 50 mL 1 × TAE buffer (40 mM Tris base, 20 mM acetic acid, 1 mM EDTA at pH 8.0) and stained with 0.6 μL of GreenSafe. Electrophoresis was performed for 40 min at 120 Volts and the gel analysed using ultraviolet (UV) light, through the Uvitec Fire-Reader system (UVITEC, Cambridge, UK).

#### 2.2.6. Characterization of Binary and Ternary Complexes

Scanning electron microscopy (SEM) was used to obtain information concerning the morphology of ternary systems at N/P ratio of 2 for PEI. The formulations were centrifuged and the pellet was recovered and suspended in an aqueous solution containing 40 μL of tungsten. The solution was placed in roundly shaped coverslip and dried overnight at room temperature. The samples were sputter coated with gold by using a K550 sputter coater (Emitech, London, UK) and a S-2700 scanning electron microscope (Hitachi, Tokyo, Japan) with an accelerating voltage of 20 kV at various magnifications was used to obtain the SEM images. 

The average size and zeta potential of developed ternary complexes were determined by Dynamic Light Scattering (DLS), at 25 °C, using a Zetasizer nano ZS device (Malvern Instruments, Worcestershire, UK). The pellet containing the complexes was suspended in 5% glucose with 1 mM NaCl. DLS techniques were performed with a He-Ne laser at 633 nm with non-invasive backscatter (NIBS) for the size and electrophoretic light scattering optics using a M3-PALS laser (Phase analysis Light Scattering) for the determination of surface charge. All experiments were analyzed through Malvern Zetasizer software v 6.34.

#### 2.2.7. Cell Culture

HeLa cells were grown in 25 cm^3^ T-flasks with Dulbecco’s Modified Eagle´s Medium with Ham’s F-12 Nutrient Mixture (DMEM-F12) with L-glutamine supplemented with 10% heat inactivated fetal bovine serum (FBS), 0.5 g/L sodium bicarbonate, 1.10 g/L HEPES and 1% (*v*/*v*) of a mixture of antibiotics composed of penicillin (100 µg/mL) and streptomycin (100 µg/mL). In a similar way, N2a cells were maintained in DMEM with 10% fetal calf serum, 1 mM glutamine, penicillin (100 µg/mL) and streptomycin (100 µg/mL). The cellular growth was promoted at 37 °C, in a humidified atmosphere containing 5% of CO_2_, until confluence was attained. Afterward cells were sub-cultivated each 7 days to maintain their exponential growth. 

#### 2.2.8. Biocompatibility Study

The biocompatibility of the complexes was evaluated on NHDF and HeLa cells by means of MTT (3-[4,5-dimethyl-thiazol-2-yl]-2,5-diphenyltetrazolium bromide) assay. This colorimetric method quantifies the metabolically active cells. HeLa cells were plated at a density of 1 × 10^4^ cells per well and grown at 37 °C in a 95% air/5% CO_2_ humidified atmosphere. The ternary complexes were applied to the well plates. After 24 h or 48 h incubation, the redox activity was assessed through the reduction of the MTT. Absorbance at 570 nm was measured using a Benchmark Microplate Reader (BioRad, Vienna, Austria). Non-transfected cells were used as negative control and ethanol treated cells were used as positive control. For the study on HeLa cells, free PTX dissolved in DMSO (stock solution of 20 µg/mL) and diluted in physiological saline solution (0.9% NaCl, *w*/*v*) to a final concentration of 5 µg/mL, and naked pDNA, PEI 25 kDa, PEI/pDNA complexes at N/P ratio of 2, PAMAM G4, PAMAM G4-PTX, used at the same amounts as for the preparation of PAMAM G4-PTX/pDNA/PEI at N/P ratio of 50:2:1 (procedure described above), were also investigated as appropriate controls. The relative cell viability (%) related to control wells was calculated by [A]_test_/[A]_control_ × 100, where [A]_test_ is the absorbance of the test sample and [A] control is the absorbance of negative control sample.

#### 2.2.9. FITC Plasmid Labeling

Plasmid DNA was stained with FITC by assembly 2 µg of pDNA, 2 μL of FITC (in sterile anhydrous dimethyl sulfoxide, 50 mg/100 µL) and 81 μL of labelling buffer (0.1 M sodium tetraborate, pH 8.5). Samples were placed under constant stirring at room temperature for 4 h and protected from light. One volume of 3 M NaCl (85 μL) and 2.5 volumes of 100% ethanol (212.5 μL) were added. Samples with stained pDNA were incubated at −20 °C overnight. Subsequently, samples were centrifuged at 4 °C for 30 min and the pellet was washed with 75% ethanol for posterior formulation.

#### 2.2.10. Cellular Uptake/Internalization

Confocal fluorescence microscopy was used to monitor the cellular uptake/internalization of the developed PAMAM G4-PTX/pDNA/PEI complexes. For this, a live cell experiment was conducted. HeLa cells were grown in μ-slide 8 well (Ibidi, Martinsried, Germany). Nucleus was stained by incubating the cancer cells with 1 µM DAPI for 10 min and pDNA was stained with FITC, as described previously. Real live transfection was visualized and analyzed using an LSM 710 confocal laser scanning microscope (Carl Zeiss SMT, Inc., Oberkochen, Germany) under 63× magnification During the study, cells were maintained at 37 °C with 5% CO_2_. 

#### 2.2.11. Determination of Cell Associated PTX

To measure the cellular internalization of PTX, HeLa or N2a cells were transfected with PAMAM G4-PTX/pDNA/PEI complexes, as described. The buffer solution used for complexes preparation (described above) and non-transfected cells treated similarly were used as reference. After 6 h of transfection, cells were washed with PBS and collected. Cells were lysed by incubation with a Triton X-100 1% solution for 30 min at 37 °C and pipetted into a black plate. The intracellular PTX levels were inferred by measuring the absorbance at 230 nm using a microplate reader (BioRad).

#### 2.2.12. Protein Quantification

The p53 protein produced by transfection of HeLa or N2a cancer cells with the developed ternary systems has been determined by p53 pan ELISA kit (Roche Applied Science, Penzberg, Germany), following the procedure described by the manufacturer. This method is based on a sandwich ELISA principle, where the investigated sample reacts with the capture antibody and peroxidase labelled detection antibody forming a stable complex. The formed colored compound is quantified by spectrophotometry and is proportional to the p53 concentration. Briefly, transfected cell lysates were prepared from detergent lysis in 1% Triton X-100 and 0.1% SDS in PBS, pH 7.4 and proteinase inhibitor cocktail. The biotin-labeled capture antibody is prebound to the streptavidin-coated microtiter plate. During a single incubation step, the p53-containing sample reacts with the capture antibody and, subsequent to the washing step, the peroxidase bound in the complex is developed by tetramethylbenzidine substrate. The protein concentration was determined by spectrophotometric measurements of the absorbance at 450 nm using a UV-Vis 1700 spectrophotometer (Shimadzu, Duisburg, Germany).

#### 2.2.13. Measurement of Caspase-3 Activity

The activity of caspase-3 was measured in HeLa or N2a cells using the ApoAlertTM Caspase-3 Colorimetric assay kit (Clontech Lab. Inc., A. Takara, Mountain View, CA, USA), following the provided instructions. Briefly, HeLa or N2a cells were serum starved and incubated with PAMAM G4-PTX/pDNA/PEI systems for 48 h. Incubation with 1 µM of staurosporine during the same period of time has been considered as positive control. The activity of caspase-3 was assayed by colorimetric detection, at 405 nm, of *p*-nitroaniline (pNA) after cleavage from the peptide substrate DEVD-pNA.

#### 2.2.14. Statistical Analysis

Normality testes were used to infer the normality of distribution of the sample data. The statistical analysis performed was *t*-test, one-way and two-way analysis of variance (ANOVA) followed by Bonferroni test. Data were compared and expressed as mean ± SEM. Data analysis was performed in GraphPad Prisma V9.0.0 software (GraphPad Software Inc., New York, NY, USA). A *p*-value below 0.05 was considered statistically significant. Additionally: * *p* < 0.05; ** *p* < 0.01; *** *p* < 0.001; **** *p* < 0.0001.

## 3. Results and Discussion 

### 3.1. Synthesis and Characterization of Multifunctional Dendrimer Conjugate 

Dendrimer-drug conjugate was synthesized by following the steps indicated in Scheme 1. PAMAM G4-PTX was characterized by means of various techniques such as ^1^H-NMR, gel permeation chromatography (GPC) and FTIR (Figure S1 available in the Supplementary Material, SM).

**Figure 1:**
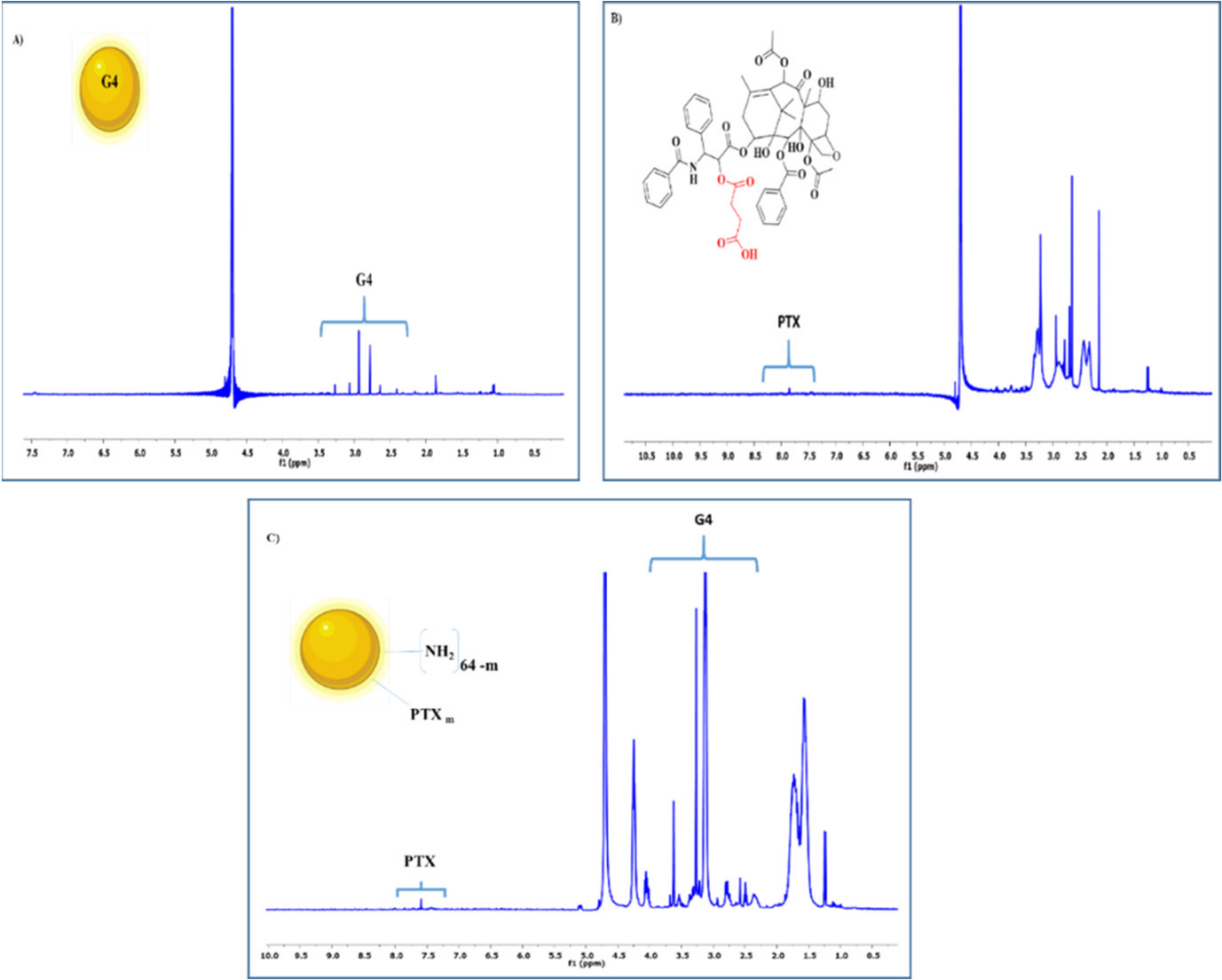
^1^H-NMR spectra of PAMAM G4 (**A**), PTX-SA (**B**), PAMAM G4-PTX (**C**).

The ^1^H-NMR spectrum of PTX-SA displayed a peak at 2.5–2.8 (δ) indicating the coupling of succinic acid to the PTX (Figure 1A). The ^1^H-NMR spectrum of PAMAM G4-PTX, showed a signal at ppm 7–8.5 (δ) corresponding to the aromatic groups of the PTX. The dendrimer protons signals were observed at ppm 1.5–3.5 (δ) (Figure 1).

The average molecular weight of PAMAM G4, PAMAM G4-PTX, was found to be 14,226, 18,771 Da, respectively (Table S1, available in SM). The GPC analysis data indicated that 4.3 molecules of PTX-SA were conjugated with one PAMAM G4 molecule. FTIR confirmed the conjugations of PTX to the dendrimer. The various spectra and some discussion on their interpretations can be consulted in SM.

### 3.2. pDNA Complexation: Binary versus Ternary Systems

The interaction of PAMAM G4-PTX and PAMAM G4-PTX/PEI with pDNA and their capacity to form nano-sized complexes was investigated by agarose gel electrophoresis. The N/P ratio considered at complex formulation step was an important parameter since it was found to deeply influence the pDNA condensation profile, as reported in the literature [10,38,39]. 

**Figure 2:**
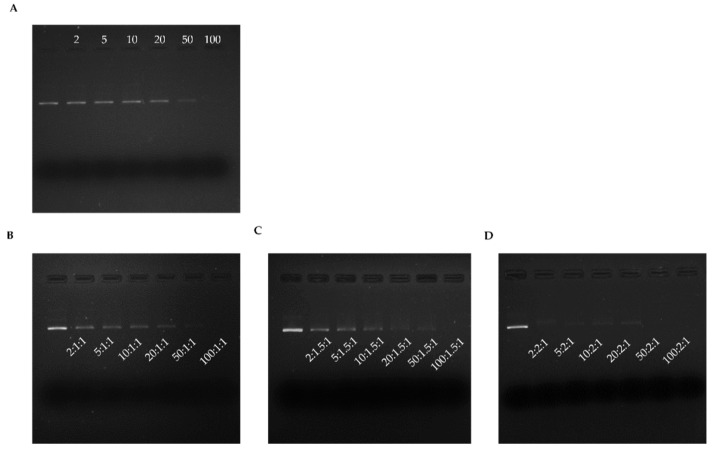
The pDNA complexation capacity of PAMAM G4-PTX/pDNA (**A**) at N/P ratios of 2, 5, 10, 20, 50, 100; PAMAM G4-PTX/pDNA/PEI (**B**) at N/P ratios of 2:1:1, 5:1:1, 10:1:1, 20:1:1, 50:1:1, 100:1:1; PAMAM G4-PTX/pDNA/PEI (**C**) at N/P ratios of 2:1.5:1, 5:1.5:1, 10:1.5:1, 20:1.5:1, 50:1.5:1, 100:1.5:1; PAMAM G4-PTX/pDNA/PEI (**D**) at N/P ratios of 2:2:1, 5:2:1, 10:2:1, 20:2:1, 50:2:1, 100:2:1 investigated by agarose gel electrophoresis. The samples were loaded at the application site at the upper end of the image and the lower end is the cathodic end. Lane 1: pDNA control.

PAMAM G4-PTX dendrimer has been considered for the complexation of pDNA. The capacity of this polymer to complex pDNA has been investigated at various N/P ratios (2, 5, 10, 20, 50 and 100) and by agarose gel electrophoresis (Figure 2A). 

This image demonstrated that PAMAM G4-PTX dendrimer is not able to complex pDNA and, thus, immobilize it for most of the ratios under investigation. At the highest N/P ratio studied (100), a strong pDNA complexation can be predicted from the almost inexistent band in the agarose gel. However, these results indicated a poor ability of the synthesized dendrimer to complex pDNA at low N/P ratios, what may compromise its use for pDNA intracellular delivery. PAMAM dendrimer interacts with pDNA by electrostatic interactions, due to the high amine content available on its surface. PAMAM G4 has 64 amines on its surface and 62 tertiary ones within its interior. However, as the drug PTX is conjugated on the surface of PAMAM G4 we may speculate that lower positive charges are present to complex pDNA and, therefore, a higher N/P ratio is needed to ensure an efficient complexation. The use of higher N/P ratios can increase the ability for pDNA complexation but can also induce higher cytotoxicity, related to the high positive charge density of PAMAM G4. Other authors reported the use of N/P ratio of 100 in the formation of PAMAM G4 dendrimer/pDNA complexes [40]. PAMAM dendrimer has been known to complex with nucleic acids forming stable nanometer complexes [41,42]. As literature suggests, the complexation process seems to be dependent on dendrimers physicochemical properties, such as, size, surface charge density, structural morphology or PAMAM generation [40,41]. Moreover, the surface amines exert a crucial role in DNA complexation and cellular internalization, while the tertiary amines can act as buffer to facilitate endosomal escape [40,43].

To improve the capacity for pDNA complexation, in this work, the highly charged cationic polymer PEI 25 kDa has been added to the PAMAM G4-PTX/pDNA complexes to form ternary systems. The aim was to efficiently neutralize the negative charges of pDNA, at lower N/P ratios. Through a screening study considering a range of N/P ratios (PAMAM G4-PTX to pDNA of 2, 5, 10, 20, 50, 100 and ratios of PEI to pDNA of 1, 1.5 and 2), the various N/P combinations of PAMAM G4-PTX/pDNA/PEI complexes were investigated. The higher ability of these PAMAM G4-PTX/pDNA/PEI to complex pDNA was confirmed (Figure 2B–D). As it is observed, pDNA can be more efficiently immobilized into the ternary complexes. The introduction of PEI significantly increased the amine positive charges what strengthen the electrostatic interaction with pDNA, improving its condensation. PEI has long been recognized by its great capacity to condense nucleic acids, mainly due to its high charge density. This polymer also exhibits an impressive endosomolytic activity from its ionization degree with pH [44,45]. The properties of PEI namely the molecular weight, the architecture of the chains or the degree of branching are fundamental parameters in determining the physicochemical characteristics of nucleic acids-PEI complexes. These properties also strongly influence both the cytotoxicity displayed by the complexes and the efficiency of cellular transfection [9,46,47].

### 3.3. Evaluation of the Properties of Binary and Ternary Systems

**Figure 3:**
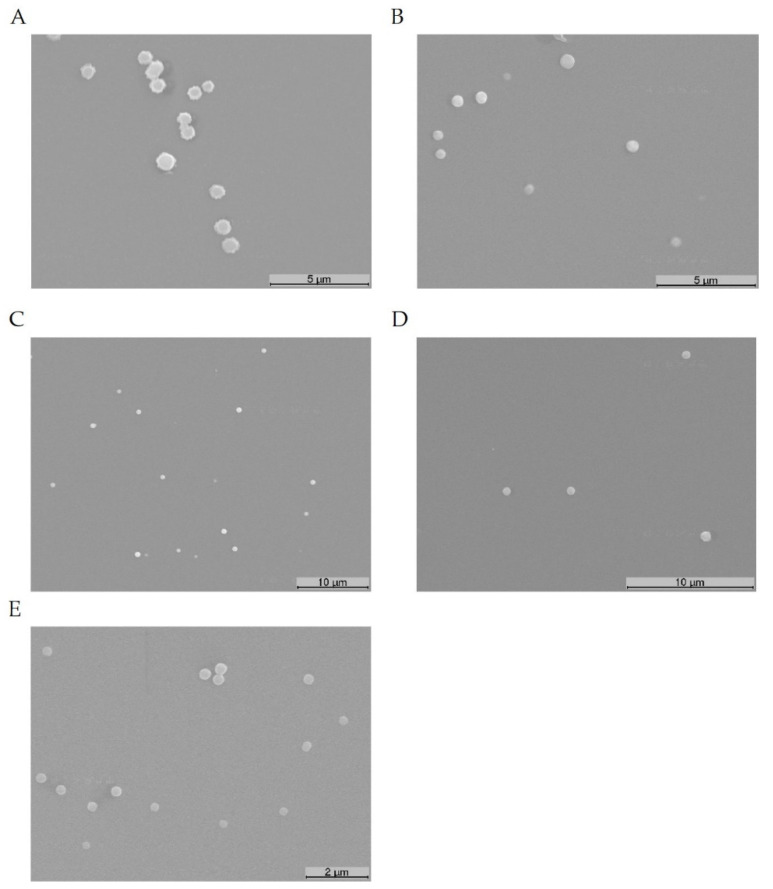
Scanning electron micrographs of PAMAM G4-PTX/pDNA/PEI complexes formulated at N/P ratios of 5:2:1 (**A**), 10:2:1 (**B**), 20:2:1 (**C**), 50:2:1 (**D**) and 100:2:1 (**E**).

The formed ternary complexes were investigated by FTIR. The various spectra can be consulted in SM (Figure S2) and are consistent with the incorporation of pDNA into the complexes. The morphology of ternary systems was studied by SEM. Figure 3 illustrates the obtained images for some of the complexes formulated at different N/P ratios. The micrographs showed that the ternary complexes exhibited a spherical or oval shape and, apparently, displayed a homogeneous structure.

The developed PAMAM G4-PTX/pDNA/PEI systems were further analyzed by DLS concerning their size, polydispersity index (PdI) and surface charges. The results are presented in Table 1. All the formed complexes displayed sizes below 500 nm, and this property varied with the N/P ratio. An increment in this parameter led to lower sizes of the systems. As the amine content increased, the interaction with pDNA became stronger leading to the condensation of the latter in a higher extent, what originated lower sized complexes. For the ternary system formed at highest N/P ratio (100:2:1), the obtained size was below 200 nm. Our results indicated that the size of ternary complexes can be easily optimized by adjusting the N/P ratio at the formation step.

The PdI for the ternary complexes has also been included in Table 1. This parameter gives information concerning the size distribution. As stated, monodisperse particles display PdI values around 0.01, while the polydisperse present values in the range of 0.5–0.7 and a PdI higher than 0.7 is indicative of broad particle size distribution [48]. In general, all the ternary complexes presented PdI values lower than 0.5 and were classified as monodisperse. The exception was found for PAMAM G4-PTX/pDNA/PEI systems formulated at lower N/P ratios (2:1:1; 5:1:1 and 10:1:1), for which some polydispersity was found.

The potential zeta of all ternary complexes has been also investigated. The results are shown in Table 1. PAMAM G4-PTX/pDNA/PEI complexes formulated at lower N/P ratios displayed negative surface charges. As the content of amines from PAMAM and PEI in-creases, the zeta potential gradually increased until a positive value was reached for the ternary system conceived at N/P of 100:1.5:1. All systems formed at N/P ratio of 2 (PEI to pDNA) exhibited positive surface charges and the values of zeta potential increased with the amines contribution from PAMAM G4-PTX (Table 1). These highest N/P ratio ternary formulations seemed to be suitable for cellular uptake purposes. Positively charged sys-tems may have a stronger capacity to interact with the highly anionic sulphated proteo-glycan molecules present at the cell surface, what facilitates cellular internalization [49,50].

The cellular uptake is a phenomenon influenced by the size, shape, surface charge and surface chemistry of the delivery system. These characteristics will dictate the mechanism of uptake, the capacity for biomolecule delivery and, lastly, the accomplished therapeutic index. In general, spherical nano-systems with low sizes (~100–200 nm) and bearing positive surface charges are preferentially captured by cells, and via clathrin-or caveolin-mediated endocytosis [50,51].

Another relevant aspect is to evaluate the properties of the developed ternary complexes in cell culture medium, as different salt concentrations and pH conditions may change the characteristics displayed by the systems. To monitor this, PAMAM G4-PTX/pDNA/PEI systems, at highest N/P ratios, were prepared as described in the experimental section and the pellet containing the complexes was suspended in DMEM, DMEM F12 and RPMI, without serum. The average particle size and zeta potential of the several PAMAM G4-PTX/pDNA/PEI complexes were determined by DLS. The results are listed in Table S2, available in SM. For all ternary systems, both the average size and zeta potential slightly increased when the complexes were immersed in DMEM, DMEM F12 and RPMI. Despite the different composition between these cell culture media, as for instance, RPMI contains a high content of phosphate ions and almost half the concentration of magnesium when compared with DMEM [52], the effect of DMEM, DMEM F12 and RPMI over the properties of PAMAM G4-PTX/pDNA/PEI complexes is barely the same. 

The developed PAMAM G4-PTX/pDNA/PEI ternary systems, namely the ones formulated at higher N/P ratios (50:2:1 and 100:2:1), offer a set of favorable properties for cellular uptake and payload intracellular delivery. Moreover, N/P ratio was identified as a tool to optimize/tailor these properties.

### 3.4. Biocompatibility Evaluation

**Figure 4:**
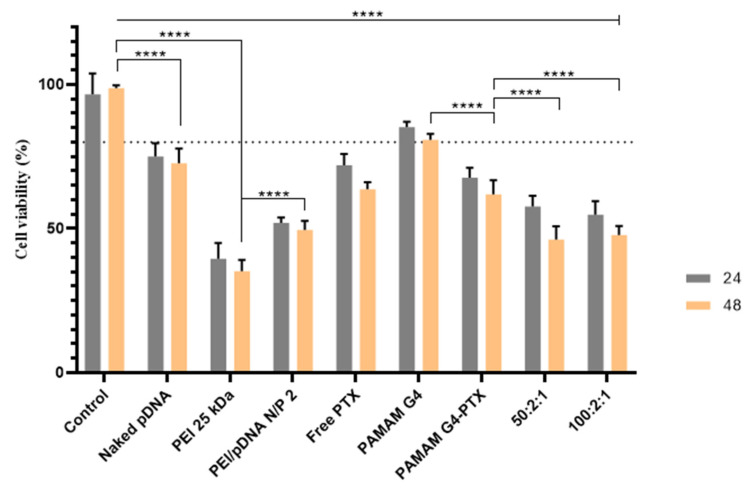
Viability of HeLa cells after 24 and 48 h incubation with naked pDNA, PEI 25 kDa, PEI/pDNA complexes at N/P ratio of 2, free PTX, PAMAM G4, PAMAM G4-PTX and PAMAM G4-PTX/pDNA/PEI complexes at N/P ratios of 50:2:1 and 100:2:1. Non-transfected cells were used as negative control for cytotoxicity. Statistical analysis was made using “one-way ANOVA” with data obtained from four independent measurements (mean ± SD, *n* = 4).

The cytotoxic profile of the developed PAMAM G4-PTX/pDNA/PEI complexes was monitored by MTT colorimetric assay on HeLa cancer cells. The cellular viability was investigated, at 24 h and 48 h, after incubation with systems prepared at N/P ratios of 50:2:1 and 100:2:1. In an attempt to discern between the biocompatibility of the complexes and the toxic effect induced by PTX and pDNA, a set of controls have been included in this experiment. The results are presented in Figure 4.

All investigated samples demonstrated to induce a significant decrease in the cellular viability, at 48 h, relative to control cells (**** *p* < 0.0001). The cytotoxic effect induced by PEI 25 kDa is more pronounced when compared to the one induced by naked pDNA or free PTX (**** *p* < 0.0001, naked pDNA or free PTX *versus* PEI 25 kDa). This fact can be attributed to the inefficient transfection of naked pDNA and to the phenomenon of high free PTX efflux from the cells [53]. On the other hand, the formation of PEI 25 kDa/pDNA complexes formulated at N/P ratio of 2 seemed to increase the viability of the systems in relation to PEI 25 kDa (**** *p* < 0.0001, PEI 25 kDa *versus* PEI/pDNA at N/P of 2, Figure 4). Concerning the dendrimer carrier itself, our study showed a less toxic effect of PAMAM G4 over control HeLa cells at 24 h, ** *p* < 0.01 for control *versus* PAMAM G4. At 48 h, the cytotoxic effect seemed to increase, **** *p* < 0.0001 for control *versus* PAMAM G4. The cytotoxic profile of PAMAM G4 could be due to the presence of multiple primary amine groups (*n* = 64). For the same reason, PEI 25 kDa showed the least cell viability. PAMAM G4 dendrimer demonstrated higher cellular viability when compared to PEI 25 kDa, indicating that it is a safer drug/gene carrier than PEI 25 kDa (**** *p* < 0.0001 for PEI 25 kDa *versus* PAMAM G4 at 48 h). The incorporation of PTX into PAMAM G4 led to a decrease of HeLa´s viability (**** *p* < 0.0001, PAMAM G4 *versus* PAMAM G4-PTX at 48 h), highlighting the toxic effect of PTX. Unexpectedly, no statistical difference was found when comparing the incubation of cancer cells with free PTX and PAMAM G4-PTX, at both 24 and 48 h. The development of ternary complexes PAMAM G4-PTX/pDNA/PEI at N/P ratio of 50:2:1 induced a higher cytotoxic effect on HeLa cells, compared with PAMAM G4-PTX conjugate (**** *p* < 0.0001, at 48 h). Therefore, the incorporation of pDNA into the complexes contributes for the decrease of the viability of cancer cells. Furthermore, both N/P ratio ternary complexes revealed to be more cytotoxic to HeLa cells than PEI/pDNA systems prepared at N/P ratio of 2 (** *p* < 0.01). The use of ternary complexes formulated at the highest ratio did not further increase the cytotoxic profile of the systems, as no statistically significant difference, at both 24 and 48 h, was found between PAMAM G4-PTX/pDNA/PEI carriers at N/P ratio of 50:2:1 and the ones prepared at 100:2:1. Altogether, the obtained results seemed to demonstrate efficient transfection mediated by the conceived ternary complexes, gene expression and p53 protein supplementation on cancer cells followed by apoptosis.

The cytotoxic profile of PAMAM G4-PTX/pDNA/PEI complexes was also studied in HeLa cells, as a function of N/P ratio. The obtained data can be consulted in SM, Figure S3. At 24 h, the ternary systems prepared at the lower N/P ratios demonstrated to be biocompatible to cancer cells, as non-statistically significant differences were found when compared to control. At 48 h, for all complexes investigated, a decrease on HeLa´s cellular viability was observed (**** *p* < 0.0001 for the comparison of control *versus* PAMAM G4-PTX/pDNA/PEI at highest N/P ratios). Moreover, it was found that the toxicity of the complexes increases with N/P ratio. For instance, statistically significant differences were obtained between the complexes formulated at N/P ratio of 10:2:1 or 20:2:1 and the ones prepared at 50:2:1 or 100:2:1 (**** *p* < 0.0001).

A cytotoxicity evaluation was also performed on NHDF cells, expressing normal p53 levels [54], to obtain deeper information regarding the biocompatibility of the developed PAMAM G4-PTX/pDNA/PEI complexes. The results are shown in SM, Figure S4. At 24 h, the ternary complexes did not induce any cytotoxicity; for complexes at various N/P ratios, the cellular viability was comparable to the one exhibited by non-transfected cells (control). At 48 h, a decrease on this parameter was observed (**** *p* < 0.0001 versus control), probably due to the increment of PTX. Despite this, all PAMAM G4-PTX/pDNA/PEI complexes seemed to exhibit acceptable levels of biocompatibility in NHDF cells (~80%). 

A comparison between the two cell lines showed the great cytotoxic effect induced by PAMAM G4-PTX/pDNA/PEI complexes in cancer cells, especially when carriers were conceived at N/P ratio of 50:2:1 or 100:2:1 (**** *p* < 0.0001, for the comparison of the cytotoxic effect induced by complexes at N/P ratio of 50:2:1 or 100:2:1 in HeLa *versus* NHDF cells). This further demonstrates the potential of the developed ternary systems in inhibiting cancer cells growth and proliferation.

### 3.5. PTX and pDNA Cellular Internalization

**Figure 5:**
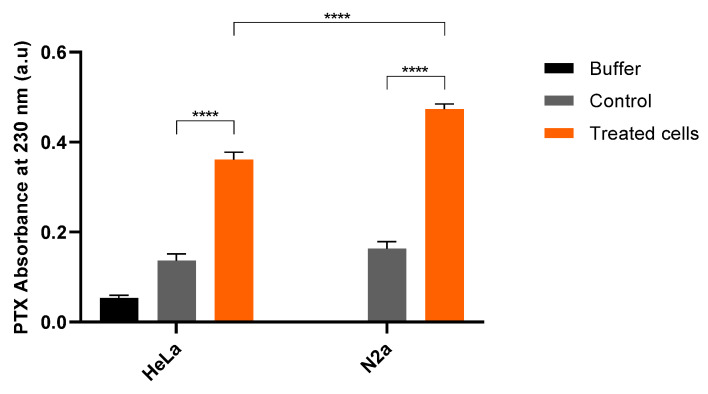
Measurement of PTX absorbance in Hela and N2a cancer cells 6 h after transfection mediated by PAMAM G4-PTX/pDNA/PEI complexes developed at N/P ratio of 50:2:1. The values were calculated with the data obtained from three independent measurements (mean ± SD, *n* = 3) and analyzed by two-way ANOVA followed by Bonferroni test.

The capacity for cellular uptake and internalization of the conceived ternary systems has been evaluated by monitoring the PTX cell-associated levels and by a microscopy study with labelled pDNA. The internalized drug into HeLa or N2a cells was inferred by measuring the absorbance of PTX after 6 h of transfection mediated by PAMAM G4-PTX/pDNA/PEI complexes at 50:2:1 ratio, as shown in Figure 5. This N/P was selected as no significant advantage was achieved by incrementing the ratio to 100:2:1, in terms of pDNA CC, surface charge or cytotoxic effect. Both the buffer solution used for the preparation of ternary complexes and non-transfected cells were used as control. HeLa and N2a transfected cells exhibited statistically significant PTX absorbance levels (**** *p* < 0.0001 *versus* non-transfected cells) giving an evidence for the presence of PTX into HeLa and N2a cells. A comparison between the two cell lines seemed to indicate higher levels of PTX in N2a cells (**** *p* < 0.0001).

**Figure 6:**
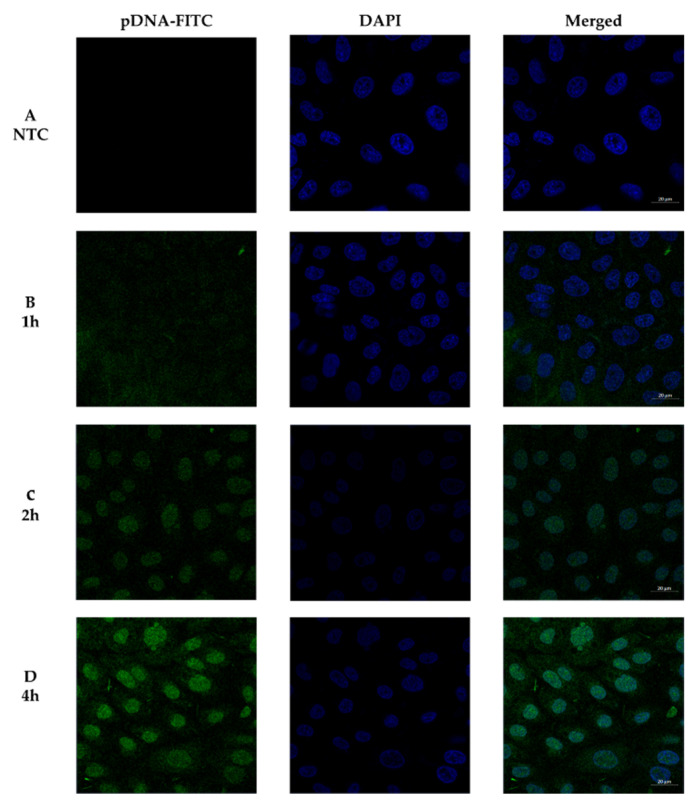
A fluorescence confocal microscopy evaluation of transfection ability and intracellular co-localization displayed by PAMAM G4-PTX/pDNA/PEI systems at 50:2:1 ratio. Nuclei are stained blue by DAPI and green represents the pDNA stained with FITC. (**A**) non-transfected cells; (**B**) 1 h, (**C**) 2 h and (**D**) 4 h of cellular transfection. Scale bar = 20 μm.

Moreover, the uptake of PAMAM G4-PTX/pDNA/PEI complexes into HeLa cells and their intracellular co-localization was visualized by fluorescence confocal microscopy. The live cell imaging study was performed at 1, 2 and 4 h of transfection. The representative microscopy images of HeLa cells are presented in Figure 6. The presented images illustrate the intracellular distribution of stained pDNA into the HeLa cells over time. As expected, there is no green fluorescence signal in non-transfected cells. After the first hour of transfection the green fluorescent dots from pDNA are barely detectable. This result suggested a poor cellular internalization after this transfection time and the need for a longer transfection time for successful uptake. After 2 h or 4 h of transfection, the extension of cellular uptake and internalization increased, and the microscopy images confirmed the pDNA internalization into cancer cells and its co-localization with the nucleus. Furthermore, a more pronounced fluorescent intensity was observed at 4 h. This study showed that PAMAM G4-PTX/pDNA/PEI systems could overcome both extracellular and intracellular barriers reaching the nucleus. The degree of carrier’s internalization increased with transfection time. Even though some of the labelled pDNA may be present in the cytoplasm of HeLa cells, at least after 4 h of transfection apparently significant levels of pDNA seemed to reach the nucleus. This phenomenon can be interpreted as a significant step towards gene expression and, consequently, protein production.

Similar results have been reported in the literature. The same dependency of transfection time was observed for PAMAM G4 dendrimer formulations for cancer treatment. In a recent study F(Fluorescein)-PAMAM G4-PEG systems were capable of A549 cells [55] and HeLa cells [56] internalization and nucleus co-localization after 4 h of transfection. Another study based on PAMAM G4 nanocarriers proved an increase in cell internalization in A549 cells over time and until 4 h [57]. A time-dependent uptake was also observed for trastuzumab-PAMAM G4 dendrimers in HER2-positive breast cancer cells [58].

### 3.6. Expression of p53 Protein

After the evidence of cellular uptake with nucleus co-localization, the capacity of the developed complexes for p53 protein expression was evaluated. The levels of p53 after transfection of HeLa or N2a cells mediated by PAMAM G4-PTX/pDNA/PEI systems at N/P ratio of 50:2.1 were determined by an ELISA assay. Untreated cells were used as control. The results are presented in Table 2. 

The complexes were able to produce a high p53 content, in contrast to the levels found for non-transfected HeLa or N2a cells. The p53 levels associated with the transfection of HeLa cells are slightly higher than the ones obtained for N2a cells. Although cervical cancer HeLa cells contain wild type p53, p53 protein was not detected. From the literature it came that besides control cells may present normal mRNA levels, the protein was not detected [10]. This fact was attributed to the relationship between E6 protein of the oncogenic mucosal-specific HPV types and p53, which resulted in p53 degradation [59].

It seemed the conceived ternary systems were able to enter the cells, protect pDNA from degradation and deliver it in the cytosol of cancer cells. It can then be hypothesized that the released genetic content, by nuclear trafficking, was able to reach the nuclear periphery. At this location, pDNA can then be directed to the nucleus by the cell endogenous import machinery, where p53 gene can be expressed [60]. Ongoing research of our group is focused on the evaluation of the anti-tumor effect mediated by the developed ternary PAMAM G4-PTX/pDNA/PEI complexes.

### 3.7. Detection of Apoptosis

Apoptosis is a physiological process that ensures normal cell function and involves cysteinyl aspartate specific proteases [61]. This cell death mechanism occurs by two main pathways, being controlled by initiator caspases (2, 8, 9 and 10) and execution caspases (3, 6 and 7). Both intrinsic and extrinsic pathways induce a set of molecular events that culminate into the activation of caspase-3. Caspase-3 acts as an executioner caspase with an important proteolytic role leading to the final steps of apoptosis [62].

**Figure 7:**
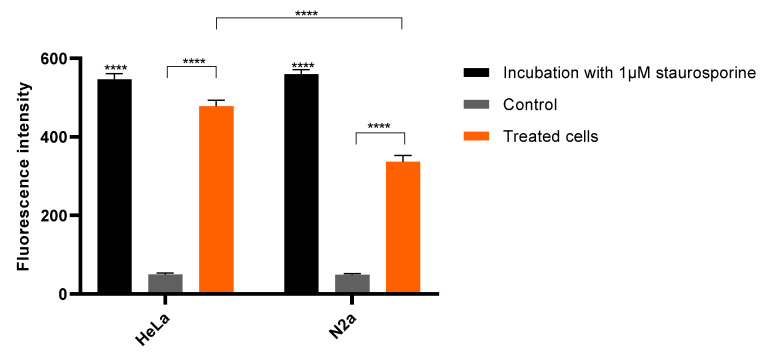
Caspase-3 activity in HeLa and N2a cancer cells after 48 h of transfection mediated by PAMAM G4-PTX/pDNA/PEI vectors at N/P ratio of 50:2:1. Incubation with 1 µM of staurosporine for 48 h was used as positive control. Data are presented as mean ± SD, *n* = 6, and analyzed by two-way ANOVA followed by Bonferroni test.

To monitor the induction of apoptosis mediated by the developed ternary complexes, at N/P ratio of 50:2:1, caspase-3 activity was evaluated in HeLa and N2a cells 48 h after transfection. The results can be consulted in Figure 7. The colorimetric assay of caspase-3 demonstrated that the caspase activity in both HeLa and N2a cells increased significantly when compared to control cells (**** *p* < 0.0001). These data seemed to be consistent with cell´s apoptosis induced from transfection with the formulated ternary systems. Moreover, a pronounced difference in the fluorescence intensity was found between the two cell lines, with higher caspase-3 levels associated with HeLa cells (**** *p* < 0.0001), what may indicate a higher extent of apoptosis in this cell line. This study gave a first insight into the capacity of the conceived ternary complexes to induce apoptosis in cancer cells, and this issue will be further examined. Future work will include the quantification of the activity of other caspases and the determination of the mitochondrial membrane potential to unravel the induced apoptosis pathway.

At this stage, we must also mention that the cellular viability data, presented in Figure 4, does not fully correlate with both the quantified p53 levels (Table 2) and the observed caspase-3 activity in HeLa cells. For this discrepancy can, probably, contribute the most common reported limitations of MTT assay, such as, lack of sensitivity, interference with cell culture medium and overestimation of cell viability [63,64].

## 4. Conclusions

To meet the crescent demand of innovative non-viral systems for cancer therapy applications, in this work we explored the capacity of both PAMAM G4-PTX dendrimer and PEI polymers to complex pDNA, forming drug/gene ternary systems. The incorporation of PEI greatly improved the pDNA CC. The co-delivery complexes have been well characterized in terms of pDNA CC, shape, mean size, surface charges and biocompatibility profile. The N/P ratio parameter was found to be an efficient tool to tailor the properties displayed by PAMAM G4-PTX/pDNA/PEI carriers. The revealed properties (as the low size and positive zeta potential) are promising for drug/gene release into cancer cells. Moreover, the systems were able of cellular uptake and both PTX and pDNA were detected in HeLa cells after their transfection with the conceived complexes. pDNA was found to co-localize with the nucleus, which resulted in the production of p53 protein. The evaluation of caspase-3 activity in cancer cells indicated apoptosis. Our results demonstrated the utility of the conception of tailor-made dendrimer ternary systems for efficient cellular transfection. These findings motivate future studies focused on the therapeutic potential of PAMAM G4-PTX/pDNA/PEI complexes, namely based on the synergistic effect, against tumoral cells.

## Figures and Tables

**Table 1:** Mean size, polydispersity index (PdI), average zeta potential and pDNA complexation capacity (CC) for PAMAM G4-PTX/pDNA/PEI complexes formulated at several N/P ratios. The values were calculated with the data obtained from three independent measurements (mean ± SD, *n* = 3).

PAMAM G4-PTX/pDNA/PEI N/P Ratio	Size (nm)	PdI	Zeta Potential (mV)	CC%
N/P 2:1:1	461 ± 35	0.63 ± 0.06	−5.05 ± 0.09	34.33 ± 3.22
N/P 5:1:1	427 ± 14	0.59 ± 0.05	−4.53 ± 0.10	41.67 ± 2.52
N/P 10:1:1	412 ± 32	0.53 ± 0.04	−4.24 ± 0.07	41.33 ± 2.31
N/P 20:1:1	409 ± 34	0.44 ± 0.03	−4.03 ± 0.13	41.33 ± 1.53
N/P 50:1:1	390 ± 26	0.38 ± 0.03	−3.12 ± 0.04	43.33 ± 1.16
N/P 100:1:1	384 ± 40	0.34 ± 0.03	−2.71 ± 0.43	45.33 ± 3.06
N/P 2:1.5:1	385 ± 24	0.38 ± 0.03	−2.14 ± 0.04	45.33 ± 1.53
N/P 5:1.5:1	383 ± 33	0.35 ± 0.02	−2.24 ± 0.08	46.67 ± 1.16
N/P 10:1.5:1	381 ± 42	0.34 ± 0.04	−2.58 ± 0.27	46.67 ± 1.53
N/P 20:1.5:1	373 ± 34	0.35 ± 0.03	−1.92 ± 0.14	47.33 ± 1.16
N/P 50:1.5:1	355 ± 23	0.33 ± 0.03	−1.09 ± 0.05	52.67 ± 2.52
N/P 100:1.5:1	334 ± 44	0.36 ± 0.04	+1.31 ± 0.27	58.33 ± 0.58
N/P 2:2:1	308 ± 23	0.38 ± 0.02	+1.22 ± 0.04	66.33 ± 1.16
N/P 5:2:1	306 ± 31	0.33 ± 0.03	+2.25 ± 0.07	69.67 ± 2.08
N/P 10:2:1	283 ± 33	0.36 ± 0.02	+4.58 ± 0.03	77.67 ± 1.16
N/P 20:2:1	259 ± 42	0.43 ± 0.03	+4.94 ± 0.10	79.33 ± 2.52
N/P 50:2:1	217 ± 31	0.35 ± 0.03	+6.99 ± 0.21	85.33 ± 1.16
N/P 100:2:1	194 ± 23	0.31 ± 0.03	+10.05 ± 0.18	89.67 ± 2.08

**Table 2:** Quantification of p53 protein levels (ng/mL) in HeLa and N2a cancer cells after 48 h of transfection mediated by PAMAM G4-PTX/pDNA/PEI complexes prepared at N/P ratio of 50:2:1. The values were calculated with the data obtained from three independent measurements (mean ± SD, *n* = 3).

		P53 Content (ng/mL)
HeLa	Control cells	0.0 ± 0
	PAMAM G4-PTX/pDNA/PEI N/P 50:2:1	482.7 ± 0.943
N2a	Control cells	40.9 ± 5.71
	PAMAM G4-PTX/pDNA/PEI N/P 50:2:1	403.2 ± 6.40

## Supplementary Materials

The following are available online at https://www.mdpi.com/article/10.3390/pharmaceutics13081256/s1, Figure S1: GPC thermogram of PAMAM G4, PAMAM G4-PTX, (A); FTIR spectra of PAMAM G4, PTX-SA, and PAMAM G4-PTX (B); Table S1: Gel Permeation chromatography data of the polymers; Figure S2. FTIR spectra (Transmittance (%) versus Wavenumber (cm-1)) of pDNA (A), PEI (B) and PAMAM G4-PTX (C) stock solutions and PAMAM G4-PTX/pDNA/PEI complexes formulated at N/P ratio of 100:2:1 (D); Figure S3: Cell viability of HeLa cells after 24 and 48 h of transfection with PAMAM G4-PTX/PEI/pDNA complexes at different N/P ratios; Figure S4: Cell viability of NHDF cells after 24 and 48 h of transfection with PAMAM G4-PTX/PEI/pDNA complexes at different N/P ratios, Table S2: Mean size, polydispersity index and average zeta potential of PAMAM G4-PTX/pDNA/PEI complexes resuspended in different cell culture media.
