# Supplementary material for: Development of Tailor-Made Dendrimer Ternary Complexes for Drug/Gene Co-Delivery in Cancer
Source: Pharmaceutics. 2021 Aug 13;13(8):1256. doi: 10.3390/pharmaceutics13081256 (PMC8401607; doi:10.3390/pharmaceutics13081256)
Supplement: Supplementary file 1 [file pharmaceutics-13-01256-s001.zip › pharmaceutics-1298487-supplementary.pdf]

pharmaceutics

MDPI

Development of Tailor-made Dendrimer Ternary Complexes for Drug/gene Co-delivery in Cancer

Ana Raquel Neves[1], Tânia Albuquerque[1], Rúben Faria[1], Paul Milan[2], Swati Biswas[2], Ângela Sousa[1] and Diana Costa

Experimental Section

Characterization of synthesized PAMAM G4-PTX dendrimer.

Gel Permeation Chromatography (GPC). The relative molecular weights of the synthesized products were determined using gel permeation chromatography (Waters Corporation, city, state abbrev USA) employing an Ultrahydrogel linear (7.8 mm ID × 300 mm × 6 μ) size exclusion column. Double distilled water was used as a mobile phase at a flow rate of 1 ml/min. The entire experiment was conducted in isocratic mode. The calibration curve was carried out using standard of shodex ranging molecular weight between 6300 to 530000 before analyzing the sample. The sample peak was analyzed by integrating and calculating using the calibration curve plotted earlier. Mn, Mw, Mp were determined, and the number of molecules attached to the dendrimers was reported.

Fourier transform infrared spectroscopy. The functional groups present in the conjugate were characterized using IR spectroscopy (Jasco-4200, city, state abbrev USA). FTIR analysis was performed using KBr pellet method [1]. The scanning range was kept from 4000 to 400 cm^{-1}. FTIR was also applied to investigate the functional groups present into the formed ternary complexes. The pellet recovered from different complexes was suspended in 10 μL of ultrapure water. The spectra were acquired by a Nicolet iS10 FTIR spectrophotometer (Thermo Scientific, Waltham, MA, USA) with an average of 120 scans, a spectral width ranging from 4000 and 600 cm-1 and a spectral resolution of 32 cm-1. The spectra of pDNA, PEI and PAMAM G4-PTX isolated samples were acquired for comparative analysis.

Results

The multifunctional dendrimer conjugate was synthesized and characterized by NMR spectroscopy and GPC. The average molecular weight of PAMAM G4, PAMAM G4-PTX, was found to be 14226, 18771 Da, respectively (Table S1). The GPC analysis data indicated that 4.3 molecules of PTX-SA were conjugated with one PAMAM G4 molecule (Figure S1 A). FTIR spectroscopy was performed to confirm the conjugations of PTX, to the dendrimer. The presence of a peak at 2927 cm^{-1} indicates the presence of NH stretch. Next, the peak at 1758 and 1250 cm^{-1} corresponds to the amide carboxyl group stretch and C-N stretch, respectively, as seen in Figure S1 B. These peaks confirmed the conjugation of PTX on the dendrimer PAMAM G4.

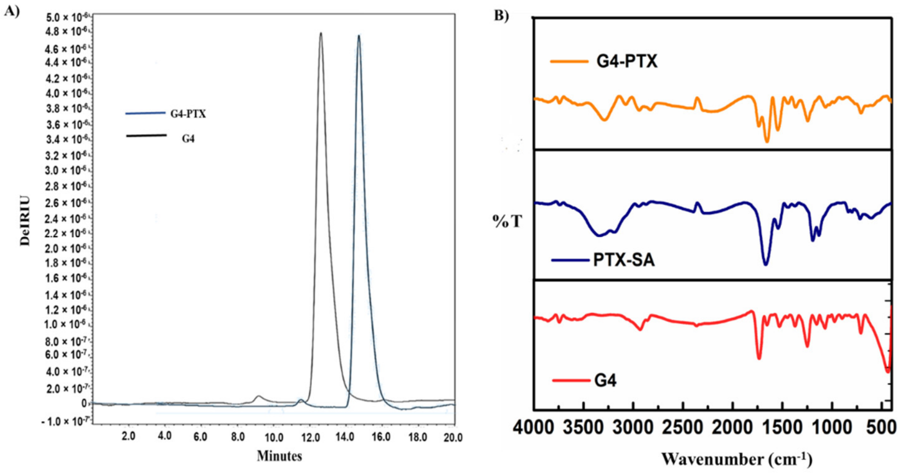

Figure S1: GPC thermogram of PAMAM G4, PAMAM G4-PTX, (A); FTIR spectra of PAMAM G4, PTX-SA, and PAMAM G4-PTX (B).

Table S1: Gel permeation chromatography data of the polymers.

SLNO	Compound name	Mw (Da)	Mn	Mp	PDI	Number of molecules attached
1	G4	14226	14191	14211	1.002	-
2	G4-PTX	18771	18580	18695	1.0124	4.3

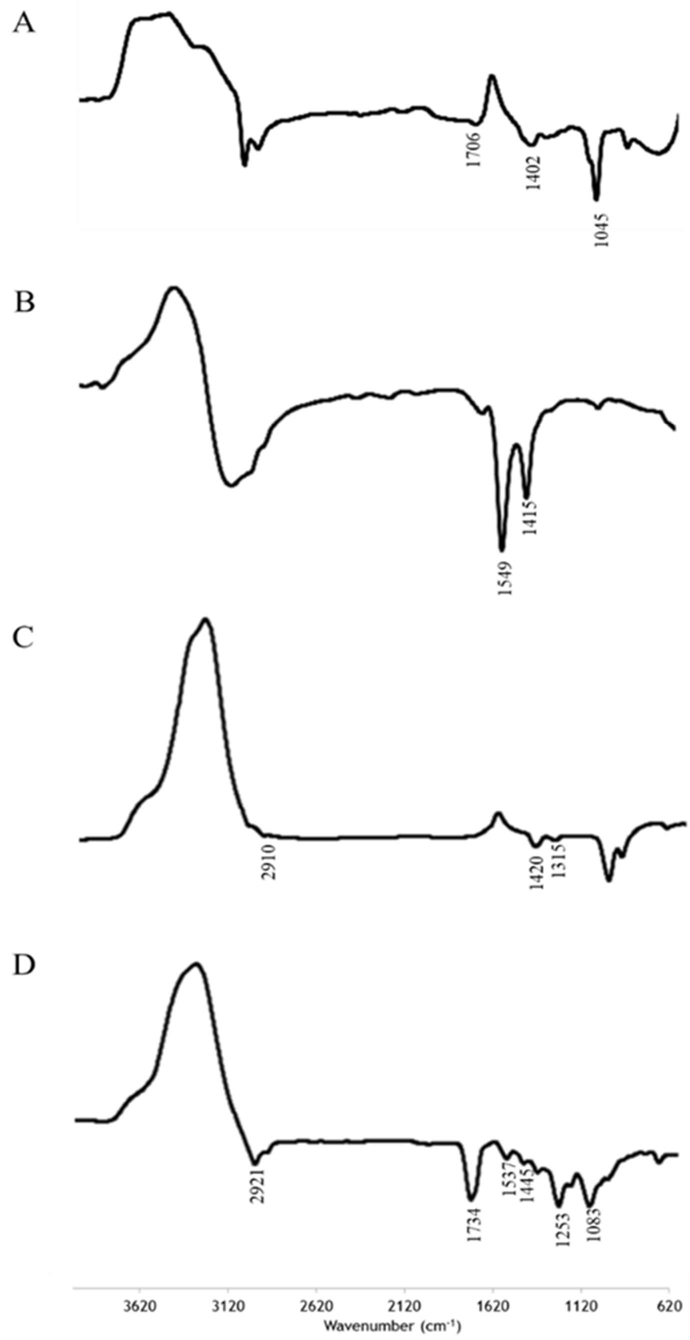

Figure S2. FTIR spectra (Transmittance (%) versus Wavenumber (cm-1)) of pDNA (A), PEI (B) and PAMAM G4-PTX (C) stock solutions and PAMAM G4-PTX/pDNA/PEI complexes formulated at N/P ratio of 100:2:1 (D).

FTIR was used to confirm the incorporation of pDNA into the ternary complexes. In Figure 3 are represented the FTIR spectra from different components and complexes. The spectrum (A) correspondent to pDNA presented peaks ranging from 1415-1706 cm^{-1} relative to nitrogen bases and a more prominent band at 1045 cm^{-1} attributed to the carbonyl stretching vibration of furanose ring. The spectrum (B) presented the peaks characteristic from PEI polymer. Two bands were observed: one at 1549 cm^{-1} representative of -NH and -NH2 groups bending vibrations and other at 1415 cm^{-1} representative of -CH scissor vibrations. PAMAM G4 dendrimer FTIR spectrum exhibited peaks from 1315-1420 cm^{-1} and a peak at 2910 cm^{-1} corresponding to the stretching vibrations of aromatic rings and -CH groups, respectively. After analyzing the spectra separately, the PAMAM G4-

PTX/pDNA/PEI interaction was examined from nanoparticles spectrum acquisition (D) in the region from 4000 to 600 cm⁻¹. The presence of a band at 1253 cm-1 attributed to the antisymmetric stretching vibration of the PO2− groups inferred about the interaction involving the deoxyribose sugar of pDNA with amine groups. Also bands at 1083 cm-1 and at 1734 cm-1 representative of carbonyl stretching vibration of furanose ring and nitrogen bases of pDNA, respectively, supported pDNA complexation. As the -CH scissor vibrations region of PEI coincided with the characteristic stretching vibrations of aromatic rings peak of PAMAM G4-PTX, other regions must be evaluated. A peak at 2921 cm-1 of -CH groups and other at 1537 cm-1 characteristic of -NH and -NH2 groups bending vibrations confirmed the presence of PAMAM G4-PTX and PEI into the ternary systems. The small shift in some values may be, probably, explained from the interaction and pDNA complexation. This process may induce changes in the exposed groups.

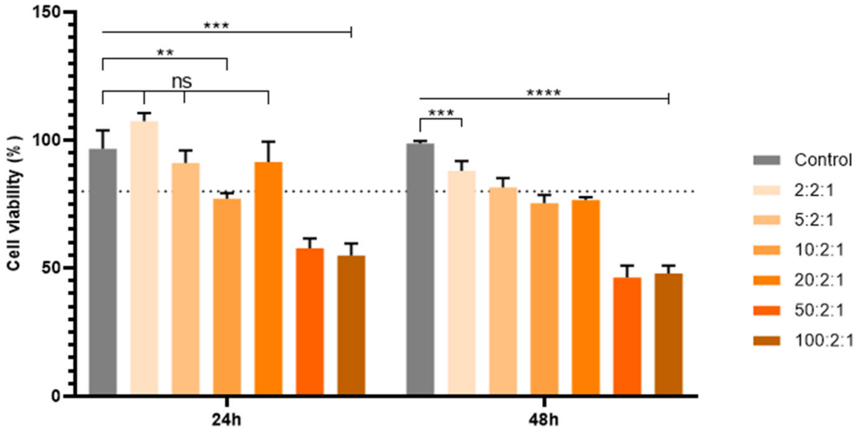

Figure S3: Cell viability of HeLa cells after 24 and 48 h of transfection with PAMAM G4-PTX/PEI/pDNA complexes at different N/P ratios. Non-transfected cells were used as negative control for cytotoxicity. Statistical analysis was made using "one-way ANOVA" with data obtained from four independent measurements (mean ± SD, n = 4). (** $p \leq 0.01$; * $p \leq 0.05$).

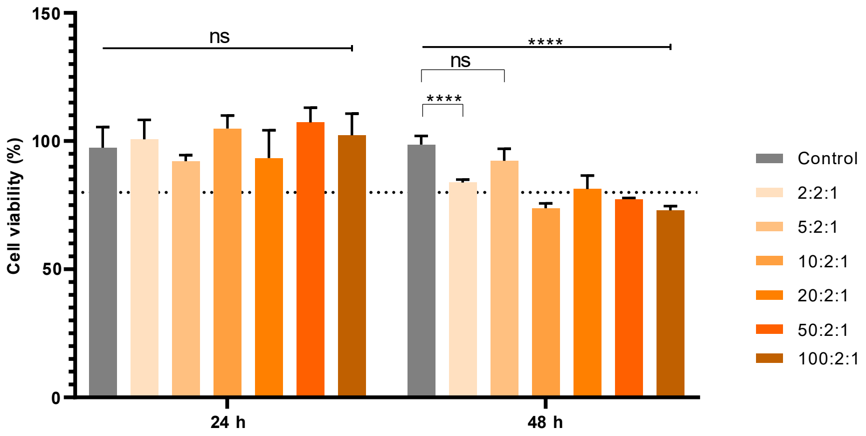

Figure S4: Cell viability of NHDF cells after 24 and 48 h of transfection with PAMAM G4-PTX/PEI/pDNA complexes at different N/P ratios. Non-transfected cells were used as negative control for cytotoxicity. Statistical analysis was made using "one-way ANOVA" with data obtained from four independent measurements (mean ± SD, n = 4). (** $p \leq 0.01$; * $p \leq 0.05$).

Table S2: Mean size, polydispersity index (PdI) and average zeta potential of PAMAM G4-PTX/PEI/pDNA complexes resuspended in three different cellular culture mediums. The values were calculated with the data obtained from three independent measurements (mean ± SD, n = 3).

**PAMAM G4-PTX/pDNA/PEI N/P Ratio**	**Size (nm)**	**PdI**	**Zeta Potential (mV)**
DMEM			
2:2:1	337 ± 22	0.45 ± 0.01	+1.60 ± 0.42
5:2:1	322 ± 51	0.49 ± 0.02	+2.34 ± 0.05
10:2:1	311 ± 30	0.41 ± 0.02	+4.90 ± 0.18
20:2:1	285 ± 46	0.42 ± 0.04	+5.01 ± 0.08
50:2:1	226 ± 49	0.37 ± 0.04	+6.79 ± 0.36
100:2:1	208 ± 45	0.39 ± 0.05	+11.01 ± 0.05
DMEM/F12			
2:2:1	343 ± 30	0.46 ± 0.03	+1.30 ± 0.02
5:2:1	325 ± 28	0.38 ± 0.03	+2.40 ±0.06
10:2:1	316 ± 38	0.49 ± 0.03	+4.98 ± 0.11
20:2:1	295 ± 63	0.42 ± 0.03	+5.11 ± 0.04
50:2:1	233 ± 47	0.43 ± 0.03	+7.09 ± 0.10
100:2:1	213 ± 30	0.40 ± 0.03	+11.56 ± 0.47
RPMI			
2:2:1	347 ± 31	0.49 ± 0.05	+2.33 ± 0.04
5:2:1	325 ± 54	0.40 ± 0.05	+5.05 ± 0.14
10:2:1	322 ± 42	0.36 ± 0.02	+5.70 ± 0.17
20:2:1	311 ± 42	0.42 ± 0.04	+6.13 ± 0.05
50:2:1	262 ± 29	0.34 ± 0.03	+7.10 ± 0.07
100:2:1	220 ± 34	0.38 ± 0.03	+12.03 ± 0.05
